# The Spatial and Temporal Influence of Cloud Cover on Satellite-Based Emergency Mapping of Earthquake Disasters

**DOI:** 10.1038/s41598-019-49008-0

**Published:** 2019-08-28

**Authors:** Tom R. Robinson, Nick Rosser, Richard J. Walters

**Affiliations:** 1 Institute for Hazard, Risk and Resilience, Department of Geography, Durham University, Durham, DH1 3LE UK; 2 Present Address: School of Geography, Politics, and Sociology, Newcastle University, Newcastle-Upon-Tyne, NE1 7RU UK; 3 COMET, Department of Earth Sciences, Durham University, Durham, DH1 3LE UK

**Keywords:** Atmospheric dynamics, Natural hazards

## Abstract

The ability to rapidly access optical satellite imagery is now an intrinsic component of managing the disaster response that follows a major earthquake. These images provide synoptic data on the impacts, extent, and intensity of damage, which is essential for mitigating further losses by feeding into the response coordination. However, whilst the efficiency of the response can be hampered when cloud cover limits image availability, spatio-temporal variations in cloud cover have never been considered as part of the design of effective disaster mapping. Here we show how annual variations in cloud cover may affect our capacity to respond rapidly throughout the year and consequently contribute to overall earthquake risk. We find that on a global scale when accounting for cloud, the worst time of year for an earthquake disaster is between June and August. During these months, 40% of the global population at risk from earthquakes are obscured from optical satellite view for >3 consecutive days. Southeastern Asia is particularly strongly affected, accounting for the majority of the population at risk from earthquakes that could be obscured by cloud in every month. Our results demonstrate the importance of the timing of earthquakes in terms of our capacity to respond effectively, highlighting the need for more intelligent design of disaster response that is not overly reliant on optical satellite imagery.

## Introduction

In the last two decades, satellite-based emergency mapping (SEM) capacities have steadily increased^1–3^. Today, most nations explicitly include SEM in their disaster response plans^4,5^, the International Charter on Space and Major Disasters^6^ has been developed to provide a unified system for space data acquisition and delivery as part of post-disaster response, and both the International Working Group on Satellite-based Emergency Mapping (IWG-SEM) and the Committee on Earth Observation Satellites Working Group on Disasters (CEOS WGDisasters) have been created to help inform SEM during disasters^7,8^. The fundamental goal of SEM is to improve the speed and effectiveness of disaster response by providing a situational overview of the extent and scale of the disaster that would otherwise be difficult to obtain from ground-based observations, particularly in remote, rural, or inaccessible locations^5,7,9,10^. UN disaster response protocols require an initial Situation Analysis within 72 hr of a disaster followed by a more detailed assessment within two weeks^11^, setting a time frame within which SEM can be most effective. Persistent cloud cover at the time of the disaster therefore presents a severe impediment when optical data are required for the post-event analysis by obscuring the affected area from optical satellite view (irrespective of how frequently imagery is captured). While this issue has previously been documented^2^, to-date no studies have quantified the effect of cloud cover despite a lack of satellite imagery being a major inhibitor to an effective disaster response and an increasing humanitarian reliance on satellite imagery^1^.

This is particularly important in the case of earthquake disasters. The majority of earthquake fatalities result from building collapse^12–15^, and whilst survival rates for victims extricated from collapsed buildings within 72 hrs can be more than 80%, this typically drops to less than 10% beyond the fourth day^12,13^. Time to rescue is therefore a key earthquake mortality risk^14^, with early emergency care potentially preventing a substantial portion of fatalities^13,16^. As a result, the IWG-SEM has proposed a series of guidelines for the rapid assessment of building damage from optical satellite imagery^17^. Any delay to identifying and undertaking a triage of relative impacts must therefore consequently result in an increase in overall earthquake disaster risk. While the use of optical satellite imagery has proved effective at rapidly identifying building damage following earthquakes^17–19^, lack of imagery due to cloud cover has previously delayed the identification of severely affected remote locations for more than a week, for example the Langtang Valley following the 2015 Nepal earthquake^20^.

Our study addresses a need to quantify the likely availability of unobscured (i.e. cloud free) optical satellite imagery to inform the design of future rapid earthquake disaster response in any given location at any given time of year. Our analysis is intended to define when imagery can be expected based on statistical analysis of historical cloud data, and so can inform decisions based upon what information disaster managers may expect to be able to access and when. The impact of our results centres around quantifying spatial and temporal variations in the resultant earthquake disaster risk, highlighting locations and timings when satellite imagery is expected to be unavailable. Our results are intended to inform the disaster response plans of national governments and humanitarian agencies based on the likely (un)availability of satellite imagery, facilitating more targeted contingency plans for specific locations and times of year.

We focus on global earthquake risk in terms of the number of people exposed to earthquake impacts annually, and explore how monthly variations in cloud cover obscure the at-risk population from satellite view. Earthquake risk is a function of hazard, in terms of the strength of ground shaking; exposure, in terms of population exposed to that shaking; and vulnerability, in terms of the exposed populations likelihood to be directly impacted by that shaking^21,22^. We also argue here that the eventual impact of an earthquake, and therefore the total earthquake risk, additionally relates to the agility and effectiveness of the ensuing response.

**Figure 1:**
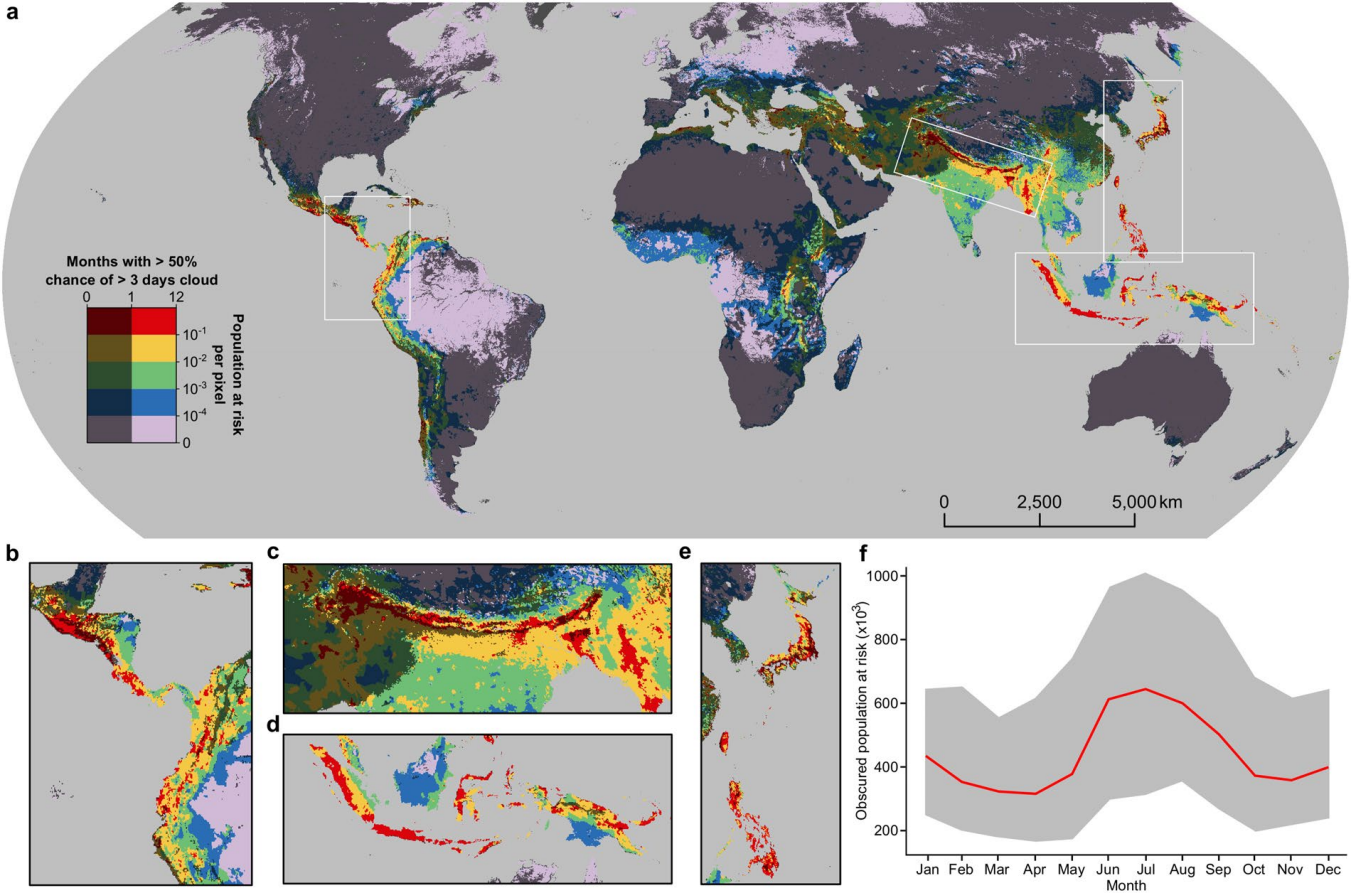
Global map of obscured earthquake risk, plotted with a Robinson projection. (**a**) Population at risk of earthquake impacts in colour overlain in dark grey with locations where the probability of >3 consecutive days cloud cover exceeds 50% in ≤1 month of the year. Areas obscured in >1 month of the year are left lighter to highlight the underlying earthquake risk in these regions. (**b**–**e**) More detailed views of Central America and northern South America (**b**), the Himalaya and surrounding regions (**c**), Indonesia and Papua New Guinea (**d**) and Japan, Taiwan and the Philippines (**e**). (**f**) Monthly variation in total global obscured population at risk showing median (red) and median ± one standard deviation (dark grey).

To quantify the number of people at risk of obscured earthquake disasters (i.e. earthquakes where cloud obscures optical satellite imagery), we first compute the number of people exposed to damaging levels of ground shaking in a single year (Figs S1 and S2). For this, we build on the Global Earthquake Activity Rate (GEAR1) model^23^, which has established the annual rate of earthquake occurrence based on combined analysis of the Global Centroid Moment Tensor catalog (1977–2004) and the Global Strain Rate Model (version 2.1) on a grid with 0.1° × 0.1° cell size. We then resample high resolution (0.008° × 0.008°) estimates of global population^24^ onto the same 0.1° × 0.1° grid. We combine both datasets with vulnerability proxies (namely Corruption Index and Human Development Index (HDI), Fig. S3) in order to assess the relative proportion of the exposed population expected to be affected; that is the total number of people likely to be killed, injured or displaced by ground shaking from an earthquake. Finally, we evaluate the probability of each pixel being obscured by cloud cover (which we define as experiencing > 3 consecutive days of cloud cover) for each month based on previous work evaluating 15 yrs (2000–2014) of twice-daily Moderate Resolution Imaging Spectroradiometer (MODIS) satellite images (Fig. S4)^25^. The principle underlying this work is to establish the global population at risk of earthquake impacts for any given year, and the total number of these at-risk people that may be invisible to optical satellites for >3 consecutive days for any given month of that year. This ultimately results in two raster outputs with a 0.1° × 0.1° cell size: (i) a global earthquake risk map showing the population at risk of earthquake disasters annually (Fig. 1); and (ii) 12 global cloud cover probability (see Methods section for details) maps that show the probability of each cell experiencing >3 consecutive days of cloud cover for each month of the year (Fig. S4). We combine these two outputs to produce a video (Supplementary Video S1) highlighting the interaction of cloud cover with earthquake risk throughout the year.

## Results

### Global risk

Our analysis indicates that each year, 1.5 million people, predominantly living in the Alpine-Himalayan belt, the Malay Archipelago, Central America, and northern South America, are at risk of earthquake impacts (Fig. 1). Of these, between 19.9% and 41.6% are obscured from optical satellite view for >3 consecutive days in any given month, with the global risk of obscured (i.e. with high probability of no useable post-event optical satellite imagery) earthquakes more than doubling between April (307,000 at-risk people obscured) and July (640,000 at-risk people obscured). The global distribution of this ‘obscured earthquake’ risk throughout the year is bimodal, with a large peak from June to September, and a second smaller peak in December and January. The former coincides with the Indian southwestern monsoon when almost all of India, the Himalaya and parts of continental Southeastern Asia have persistent cloud cover, while the latter corresponds to the rainy season in the Malay Archipelago (Supplementary Video S1). Flooding and landslides in these locations at these times of year are also common^26^, further exacerbating earthquake disaster risk through increased hazard during these periods.

**Figure 2:**
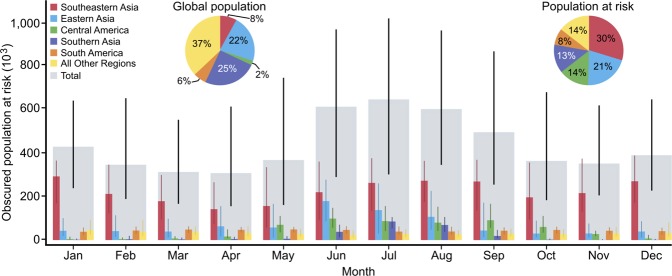
Spatial and temporal distribution of obscured population at risk grouped by UN global sub-regions for each month of the year. The inset charts show each region’s percentage of the total global population and percentage of the total annual population at risk from earthquakes irrespective of cloud cover. Vertical lines show the median ± one standard deviation.

Globally, earthquake risk is concentrated in Southeastern Asia (defined according to the UN global sub-regions), which accounts for 8.4% of world population but 29.7% (~450,000) of the global annual population at risk of earthquake disasters (Fig. 2). The number of these at-risk people obscured from satellite view varies by a factor of two during the year, with a maximum of ~300,000 in January and a minimum of ~150,000 in April. This highlights how the timing of an earthquake can potentially limit our capacity to respond effectively; in this region SEM will be significantly less effective at providing a synoptic overview of earthquake impacts within 72 hrs in January than in April. Earthquakes in Southeastern Asia in January are therefore likely to present a far greater challenge for responders, and thus a greater risk, than comparable earthquakes only three months later in April.

While Southeastern Asia has the largest obscured population at risk all year (Fig. 2), its relative global share varies from a maximum of 69.2% in December to a minimum of 36.3% in June. This minimum coincides with the onset of the Indian southwestern monsoon in Eastern and Southern Asia and the rainy season in Central America. These meso-climatic system-affected regions show the largest relative changes in obscuration throughout the year, with Central America experiencing a 21.9-fold increase in obscured at-risk population between January (~4,500 at-risk people) and June (~99,000 at-risk people), while Southern Asia undergoes a 166-fold decrease between July (~85,500 at-risk people) and November (~500 at-risk people). Such large relative changes in obscuration highlight the importance of the temporal variability in the utility of SEM for rapid emergency response and the need to account for these variations in pre-disaster response planning and design.

### National risk

**Figure 3:**
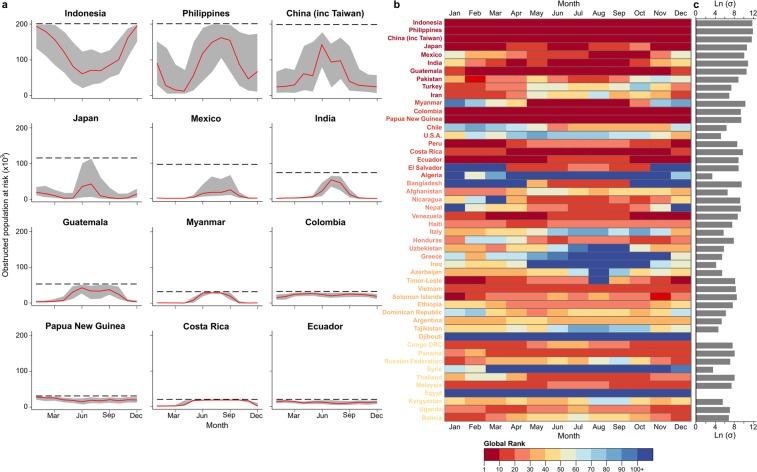
Annual variation in risk and global rank. (**a**) Monthly variation in total obscured population at risk for selected high-risk countries, showing mean number (red line), mean ± one standard deviation (dark grey), and total annual population at risk of earthquake impacts (dashed black line). (**b**) Monthly variation in global rank due to cloud cover for the 50 countries with the largest number of people at risk of earthquake disasters annually irrespective of cloud cover, listed in descending order with the largest population at the top. (**c**) Annual variation in obscured population at risk for each country expressed as the natural logarithm of its standard deviation.

Annual variability in obscuration is even more extreme at the national-level, with Indonesia, the Philippines, and India in particular experiencing large variations, although some countries, such as Colombia and Papua New Guinea, have comparatively constant obscured at-risk populations throughout the year (Fig. 3). By ranking countries in terms of their monthly population at risk from obscured earthquakes, it is possible to identify the locations and months where cloud cover may have the largest effect on rapid emergency mapping. Indonesia, China, and the Philippines have the largest absolute variation in obscured population, but are consistently the top 3 nations for total obscured population throughout the year (Fig. 3). Comparatively, India is the fourth highest nation in July and August but falls as low as 43^rd^ by December, while Bangladesh ranges from a rank high of 11^th^ in July to a rank low of 136^th^ in December. These variations in global rank are even more stark when comparing a nation’s rank with and without cloud (Fig. 3b). For example, Algeria has the 20^th^ largest annual population at risk of earthquakes, but is outside the top 100 for obscured population at risk for nine months of the year. Conversely, Panama ranks 42^nd^ for total population at risk, but peaks with the 11^th^ largest obscured population at risk in October and November. This wide variation in rank highlights that no country can be considered the ‘most at-risk’ location for obscured earthquakes, as this undesirable title varies significantly throughout the year.

### Earthquake response times

**Figure 4:**
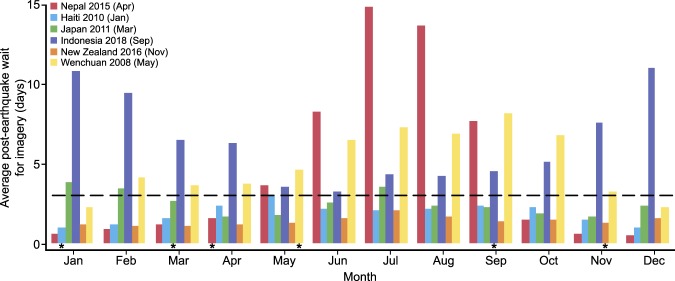
Average number of days wait for cloud free conditions in each month for historic earthquake disasters. The month each earthquake occurred is shown by the * symbol below the corresponding column. Dashed black line shows the 3 day (72 hrs) threshold used in this study.

Our analysis allows us to test how previous earthquake disasters may have been affected by cloud cover had they occurred in different months of the year. Here, we examine six recent earthquake events in which satellite data proved vital to the response: Nepal in April 2015; Haiti in January 2010; Japan in March 2011; Indonesia (Sulawesi) in September 2018; New Zealand (Kaikōura) in November 2016; and Wenchuan in May 2008. The results highlight how the timing of these six earthquakes could have influenced the ability to collect cloud free optical satellite imagery across the affected area (Fig. 4). Notably, each of these events occurred when the average number of days wait for cloud free imagery across the affected area were at, or close to, the minimum, hence allowing satellite imagery to play an important role in the response. However, responders to the Nepal 2015, Indonesia 2018, and Wenchuan 2008 earthquakes may have experienced significant delays in obtaining cloud free imagery had the earthquake occurred at a different time of year. The largest variation is for the Nepal earthquake, where the average wait time in April, when the disaster occurred, is 1.6 days, but by July has increased to 14.8 days. For the Indonesian earthquake in September the average wait time is 4.8 days but had the event occurred in December, it would have been 11.0 days. Similarly, in Wenchuan in May, when the 2008 earthquake occurred, the average wait is 4.6 days but would have been 8.2 days in September. Had these events occurred in months with the longest wait times for cloud free imagery, it is probable that optical satellite imagery would have played a very different role, if any at all, in the response.

**Figure 5:**
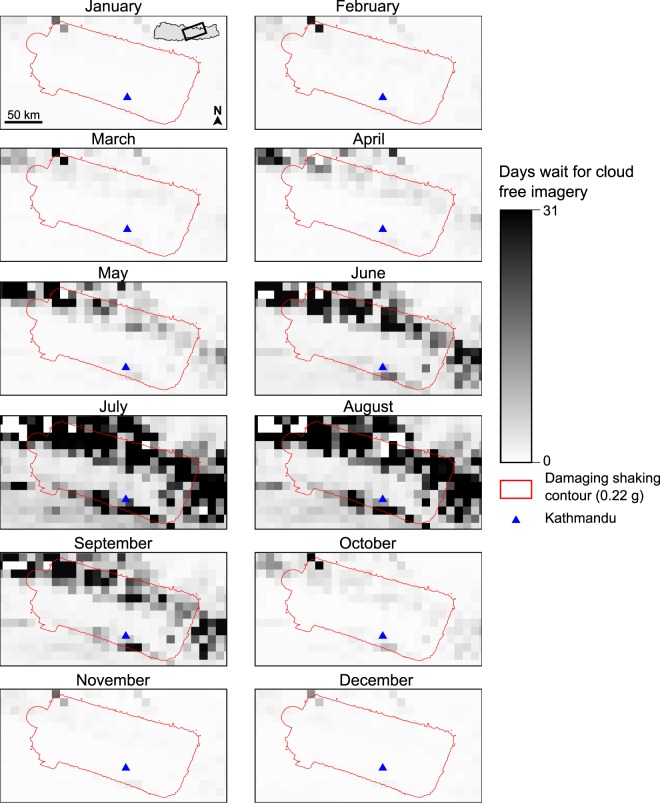
Average number of days wait for cloud free imagery in central Nepal. Monthly variation in wait time for cloud free imagery over the area affected by damaging shaking during the April 2015 Nepal earthquake.

The only comprehensive timeline of both available satellite imagery and cloud effects published for these six events is for the Nepal 2015 earthquake^27^, which broadly validates our results. While our work suggests an average wait across the affected region of Nepal in April of 1.6 days, the longest wait in an individual cell is 16.8 days over the mountains in the north-west of the area, while over Kathmandu and the low-lying areas to the south wait times are just 1–2 days (Fig. 5). Following the earthquake, the first cloud-free imagery became available over Kathmandu after 2 days, followed by the low-lying regions to the south after 4 days^27^. However, complete cloud free imagery of the entire affected area took 13 days to capture, when imagery of the ground was successfully captured over the mountains in the north-west. While the average wait time for the area affected by the Nepal earthquake is highest in July (Fig. 4), it is significant that much of the mountainous area in the north of the region is not expected to have any cloud free days between June and September, during the monsoon. Consequently, had this earthquake occurred at the start of June, it may have taken until October before cloud free imagery for the entire affected area was available (Fig. 5). Given that these mountainous northern regions experienced some of the most significant damage in the 2015 earthquake^20,28^, this would have presented a severe impediment to the emergency mapping. This highlights the drawbacks of relying on optical satellite imagery for emergency response in Nepal during the monsoon and the need for viable alternatives.

## Discussion

While results from our study do not causally link cloud cover with higher rates of earthquake fatalities, or SEM with lower rates of fatalities, they nonetheless highlight how the timing of an earthquake can affect SEM activities and the humanitarian disaster response. A reliance on optical satellite imagery alone to inform rapid emergency response may therefore exacerbate earthquake risk globally, highlighting the need to develop other means of rapidly assessing impacts. Recent advances in coherence mapping with Synthetic Aperture Radar (SAR) satellites may present a viable solution, as this has the ability to ‘see’ through cloud as well as identify buildings that have undergone ‘pancake-style’ collapse, which is difficult in optical imagery^2,17^. While there have been several notable developments in, and successful applications of, SEM using SAR^19,29–31^, at present there remain key shortcomings: routine operational revisit times for most SAR-capable satellites are longer than 72 hrs^32^; recent pre-event imagery must be available in the same configuration as post-event imagery; pixel sizes are typically much larger than optical imagery; and processing and interpreting the imagery requires a high degree of skill, precluding the use of public crowd-sourced mapping^1,2,29^. Continued increases in the number of SAR-capable satellites, and in particular the number of multi-satellite constellations such as Sentinel-1 and COSMO-Skymed, continue to reduce this latency time^33^. In addition, shorter post-disaster latency of image acquisition can also be achieved by emergency tasking e.g. through activation of the International Charter on Space and Major Disasters^6^, while an increased number of users and further development of automated analysis techniques will also likely decrease the time between image capture and map production^1^. For example, for our highest obscured risk locations of Indonesia and the Philippines, emergency tasking of satellite constellations such as COSMO-Skymed can enable SAR imagery to be acquired within a few hours of earthquake occurrence^34^. However, it should also be noted that emergency tasking is not always possible, and that different SAR missions and acquisition modes have differing areal coverage^32^, meaning that data will not always be suitable for assessing earthquake damage and impacts on the largest (>~100 km^2^) scales following major (M > 7.5) earthquakes. In the interim, a concentrated reliance on optical-based SEM for earthquake emergency response may only be appropriate in countries like Turkey and Iran, where limited cloud cover means optical satellites present a reliable tool for rapid assessment year-round. Elsewhere, emergency response plans need to be designed to recognise that optical SEM will be reliable only at certain times of year and so planning for alternative means of impact assessment, including SAR and non-space-based approaches, is essential during these times.

## Methods

### Global earthquake risk mapping

To establish global earthquake hazard, we use the GEAR1 global seismicity model^23^, which provides estimates of the annual number of earthquakes occurring above set magnitude thresholds for each 0.1° × 0.1° cell and has been recently validated by comparison with globally observed earthquakes^35^. However, because large magnitude earthquakes can cause impacts across areas larger than 0.1° × 0.1°, we convert this to the likelihood of damaging shaking occurring in each cell. This allows for direct comparison across a range of earthquake magnitudes and accounts for impacts resulting from earthquakes located in neighbouring cells.

From the GEAR1 model, we derive the annual frequency of earthquakes per pixel for magnitude bins M 6.0–6.5, M 6.5–7.0, M 7.0–7.5, M 7.5–8.0, M 8.0–8.5, and M > 8.5. We then use these outputs to estimate the annual frequency of each pixel experiencing damaging shaking. For this, we consider a damaging shaking threshold of 0.22 g, as empirical fragility functions for multiple global building types suggest this is the minimum shaking required to initiate collapse in most types of structure^36^, and the large majority of earthquake casualties result from building collapse^12–15^. To estimate the total number of pixels exceeding 0.22 g of shaking for each magnitude range, we compare the results from 15 different ground motion prediction equations (GMPEs)^37–45^ for various fault mechanisms, locations, and tectonic regimes and average the results to derive a single radial distance for each magnitude bin. For simplicity, azimuthal variations in this distance depending on earthquake size or type are not considered. Site effects are ignored due to the need for a global threshold and focal depths are held constant at 15 km. The distances for each magnitude bin are: M 6.0–6.5 = 19.7 km; M 6.5–7.0 = 24.3 km; M 7.0–7.5 = 35.4 km; M 7.5–8.0 = 53.0 km; M 8.0–8.5 = 77.2 km; and M > 8.5 = 107.9 km (Fig. S1). For each cell, the sum of all earthquakes within the corresponding radial distance is calculated for each magnitude bin, and a total sum for all magnitude bins gives the expected total annual frequency of damaging shaking for each cell (Fig. S2).

Annual population exposure to damaging shaking is calculated from the 2015 Gridded Population of the World version 4 (GPWv4)^24^ by multiplying the total population in each cell by the annual frequency of shaking (Fig. S2). Many factors control human vulnerability to earthquakes, but at the global scale corruption and HDI have been shown to be indicative of population fragility to earthquakes^46–48^, with lower levels of development and higher levels of corruption individually associated with larger earthquake impacts. To evaluate the vulnerability of the exposed population in each cell to earthquake impacts, we combine national-level corruption scores^49^ with subnational HDI^50^ from the same year as the population data (2015) to derive a single vulnerability proxy (Fig. S3). Corruption is scored out of 100 while HDI is scored out of 1, with lower values representing less development and higher corruption. We combine these such that

1 $$Vulnerability=1-(\frac{Corruption}{100}\times HDI)$$

with final vulnerability scores ranging between 0 and 1, representing the fraction of the exposed population likely to be directly impacted by an earthquake. We then multiply the annual population exposed to damaging shaking by our vulnerability proxy to derive earthquake risk (i.e. the total number of people likely to be impacted by earthquakes each year) for each cell. We then derive total global, sub-regional, and national (based on UN definitions) population at-risk by summing for all relevant cells.

### Probability of obscuration

We consider obscuration to the be the inability to see the ground with optical satellites for >3 consecutive days due to cloud cover, where each cell has >50% cloud. We consider cloud cover of >3 consecutive days to correspond with the 72 hr time-frame dictated by UN disaster response protocols^11,27^ and the rapid decrease in survival rates for trapped victims beyond this time^13^. We do not include estimates of seasonal changes in daylight through the year in our calculations as most optical satellites are on sun-synchronous orbits that are only affected by seasonal variations in daylight at high (>65°) latitudes, where both population and earthquake hazard are low (Figs 1 and S2). To derive the probability of each cell experiencing >3 consecutive days of cloud cover in each month of the year, we use outputs from previous work using 15 yrs of MODIS satellite data^25^. This previous work calculated the mean number of days each cell was affected by >50% cloud cover for each month of the year, as well as the standard deviation for each month across the 15 yrs. This allows us to calculate the probability of cloud cover on any given day in each month for each cell, and thus the probability of >3 consecutive days cloud cover (Fig. S4):

2 $$P({1}\,day\,cloud)=Mean\,cloudy\,days\,per\,month/Number\,of\,days\,in\,the\,month$$

3 $$P(\, > \,n\,consecutive\,days\,cloud)=P{({1}daycloud)}^{n}$$

We assume here that the likelihood of cloud cover each day is independent of the previous day’s cloudiness. The standard deviation of cloudy days for each month is used to derive upper and lower bounds on the daily probability of cloud cover, representing intra-monthly variation. We define a cell as being obscured if, for a given month, it has >50% probability of >3 consecutive days cloud cover, and sum for the total at-risk population within these cells to evaluate the at-risk population obscured from satellite view.

### Cloud cover in past earthquake disasters

For each event, we evaluate the average number of days wait for cloud free conditions within the area that experienced damaging shaking (>0.22 g) for each month in order to examine how the timing of the event may have changed the use of imagery in the response. To calculate the average number of days wait for each cell globally, we set the probability in Equation 3 constant at 50% and solve for the number of days (n). We then take the monthly mean and maximum values of all cells contained within the damaging shaking zone, using peak ground acceleration (PGA) data for each event from the USGS.

## Supplementary information

Monthly obscured earthquake risk

Supplementary Information

## Data Availability

All data used in this study are available from open source repositories. GEAR1^23^ source code and data files are available from https://pubs.geoscienceworld.org/ssa/bssa/article/105/5/2538/332070/gear1-a-global-earthquake-activity-rate-model. Population data^24^ are available at 10.7927/H4X63JVC. National corruption scores^49^ are available from Transparency International at https://www.transparency.org/cpi2015#downloads. Sub-national human development scores^50^ are available from the United Nations Development Programme (UNDP) at https://hdi.globaldatalab.org/areadata/. MODIS cloud data^25^ are available from https://figshare.com/articles/MODIS_Cloud_Climatology/1531955 and 10.6084/m9.figshare.1531955.
