# Supplementary material for: The Spatial and Temporal Influence of Cloud Cover on Satellite-Based Emergency Mapping of Earthquake Disasters
Source: Sci Rep. 2019 Aug 28;9:12455. doi: 10.1038/s41598-019-49008-0 (PMC6713744; doi:10.1038/s41598-019-49008-0)
Supplement: Supplementary file 2 — Supplementary Information [file 41598_2019_49008_MOESM2_ESM.pdf]

Supplementary Information for:

THE SPATIAL AND TEMPORAL INFLUENCE OF CLOUD COVER ON SATELLITE-BASED EMERGENCY MAPPING OF EARTHQUAKE DISASTERS

Tom R. ROBINSON[1,2*,] Nick ROSSER[1], Richard J. WALTERS[3]

[1]Institute for Hazard, Risk and Resilience, Department of Geography, Durham University, Durham, DH1 3LE, UK.

[2]Now at: School of Geography, Politics, and Sociology, Newcastle University, Newcastle-upon-Tyne, NE7 1RU, UK.

[3]COMET, Department of Earth Sciences, Durham University, Durham, DH1 3LE, UK.

*thomas.robinson@newcastle.ac.uk

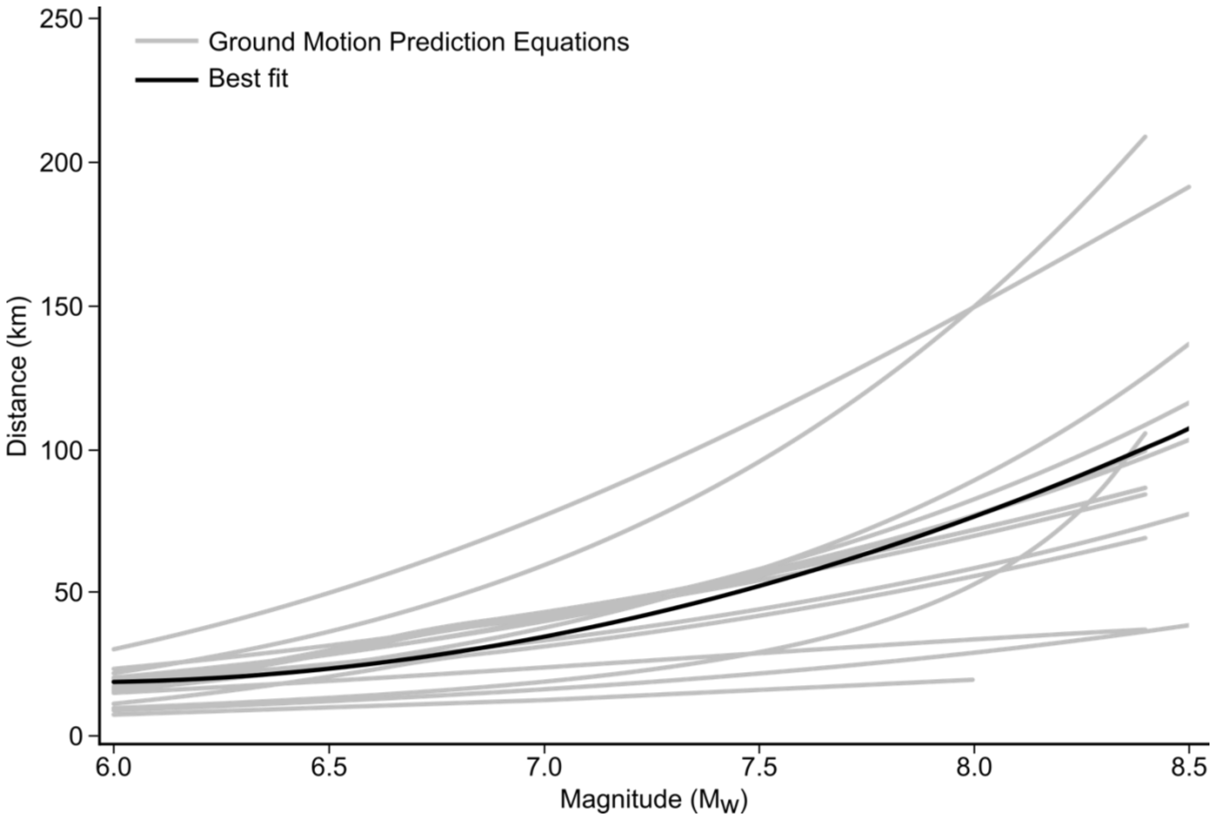

Figure S1. Magnitude-“damaging shaking” scaling. Distance from earthquake epicentre to 0.22 g shaking contour for different earthquake magnitudes calculated from various different ground motion prediction equations[1–9]. Grey lines show different GMPEs considered; Black line shows the best fit line used in this study.

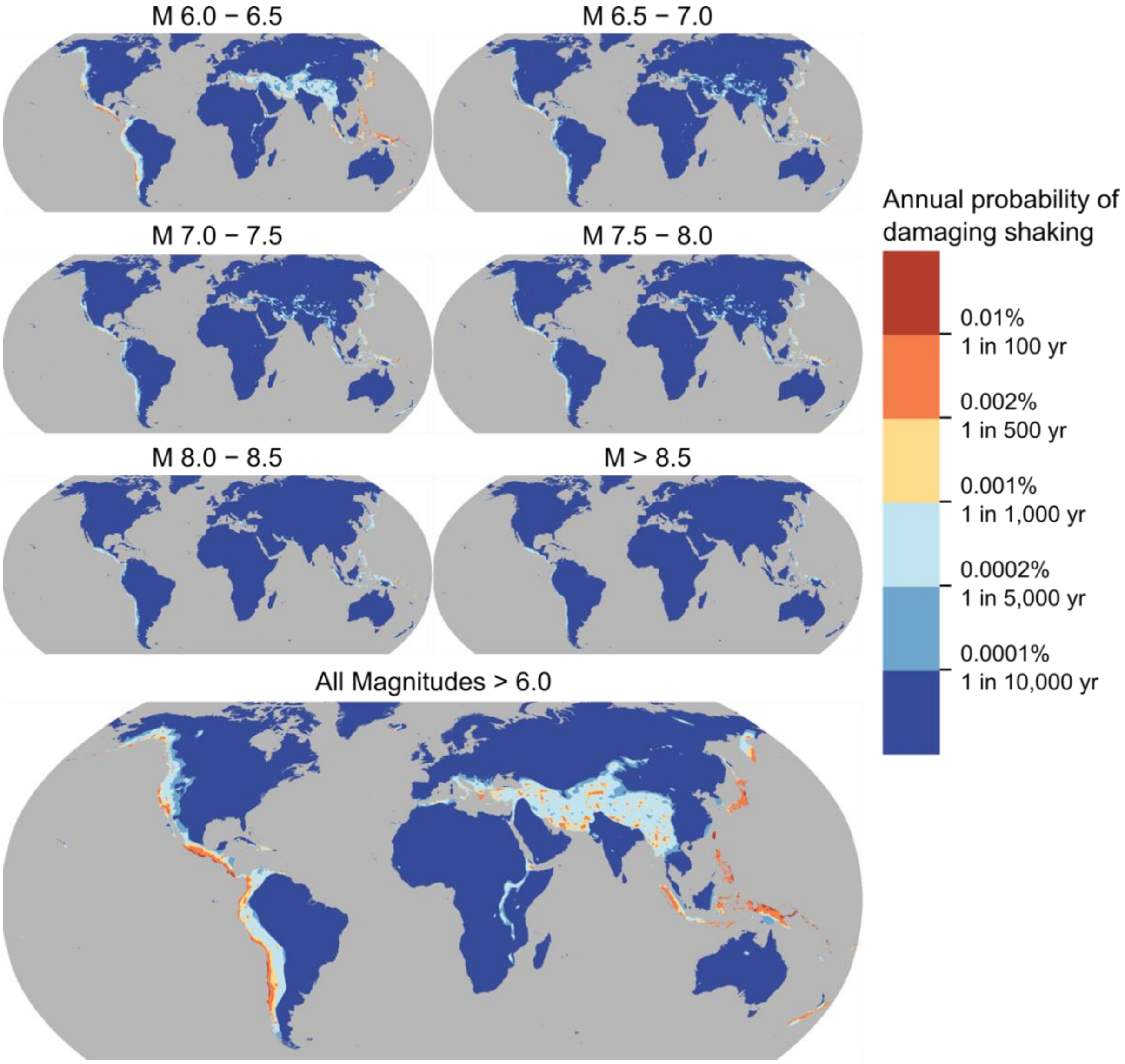

Figure S2. Probability of damaging shaking. Global maps of the annual probability of each $0.1^{o} \times 0.1^{o}$ cell experiencing ≥ 0.22 g of ground shaking based on the GEAR1 model[10] for variouis earthquake magnitude ranges.

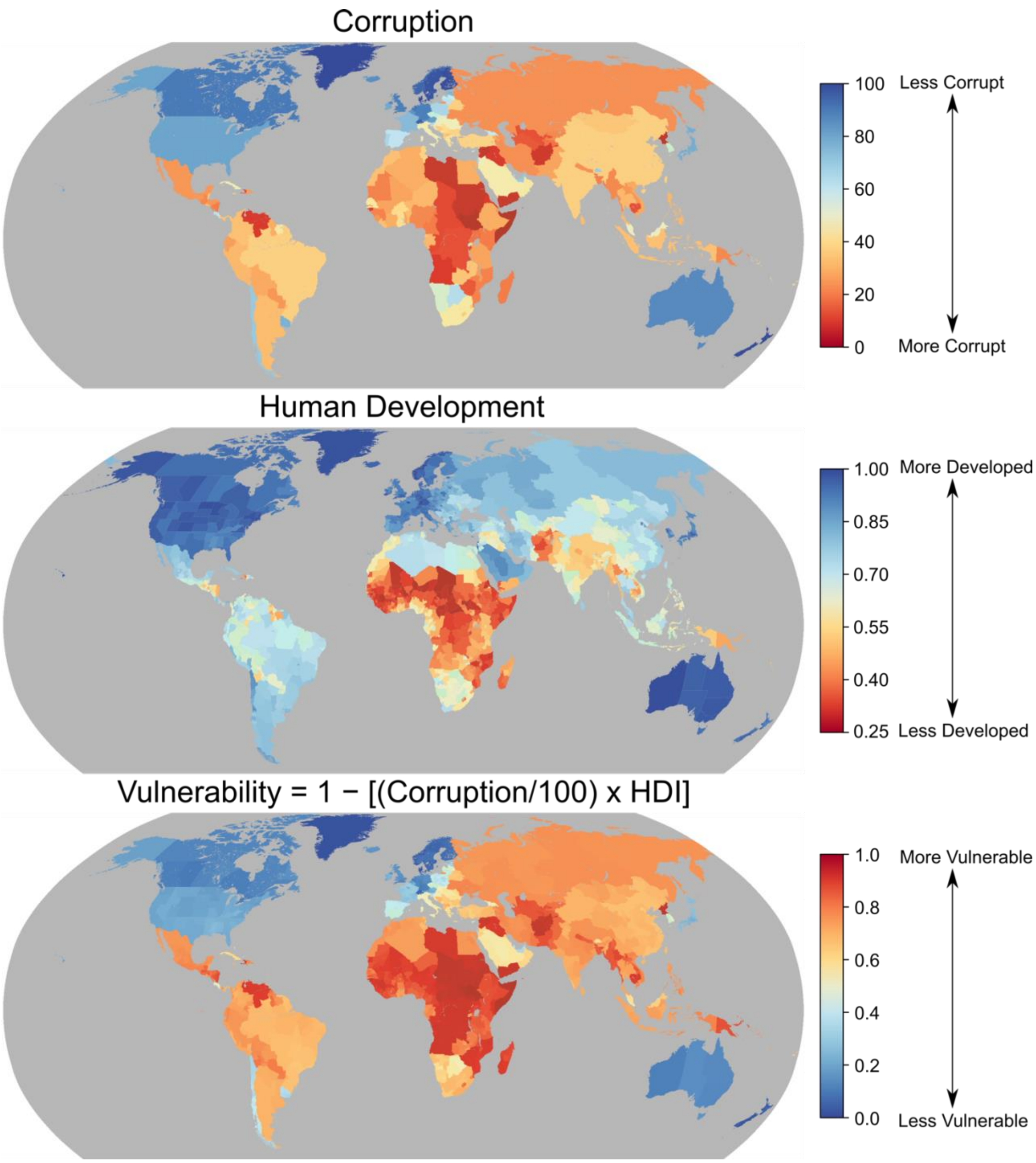

Figure S3. Earthquake vulnerability proxies. Global maps of national corruption scores[11], subnational human development scores[12], and the derived subnational population vulnerability to earthquakes. The vulnerability score represents the fraction of the exposed population likely to be directly impacted by an earthquake.

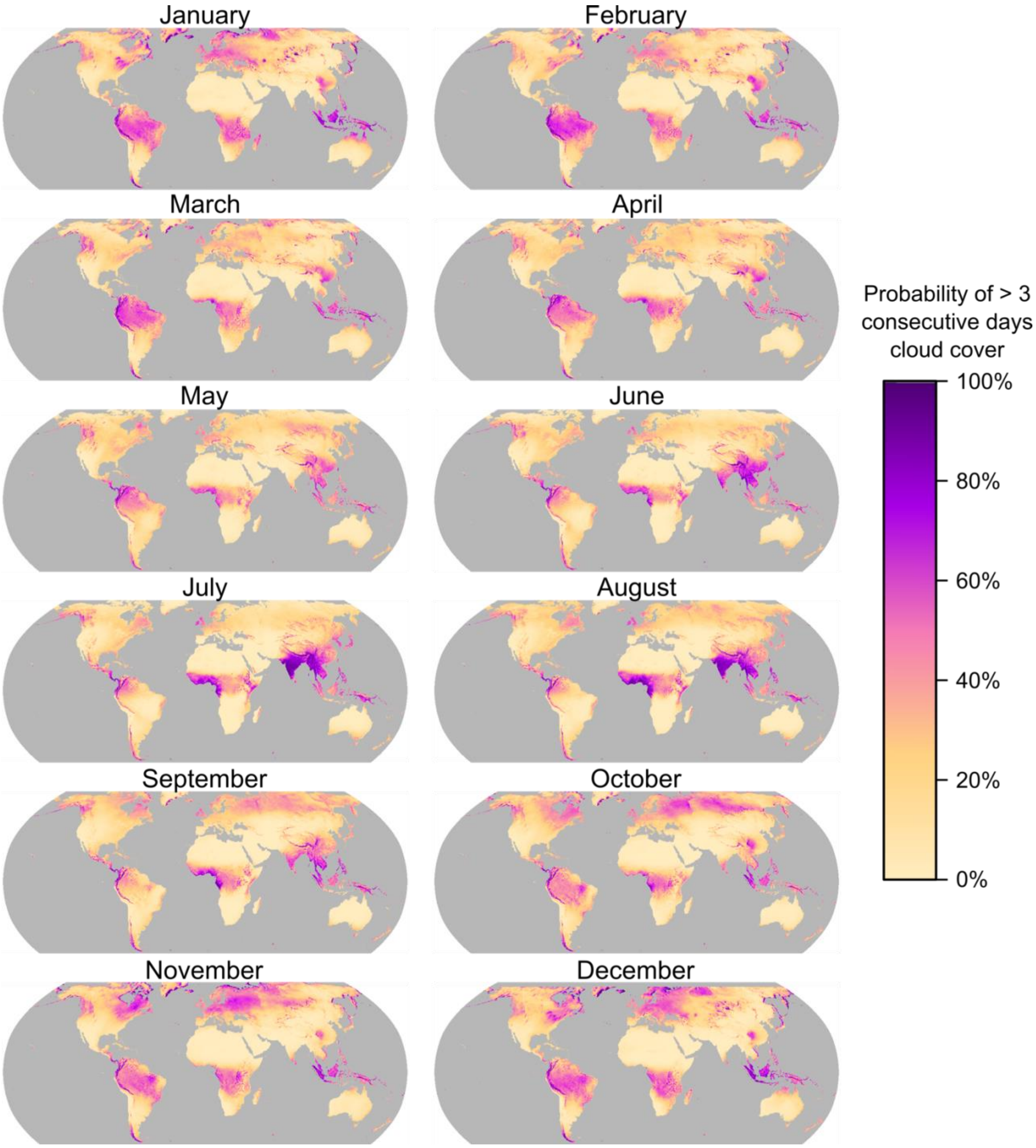

Figure S4. Probability of > 3 consecutive days of cloud cover for each month of the year derived from 15 yrs twice daily MODIS satellite observations from Wilson and Jetz[13].

REFERENCES

1. Kanno, T., Narita, A., Morikawa, N., Fujiwara, H. & Fukushima, Y. A New Attenuation Relation for Strong Ground Motion in Japan Based on Recorded Data.

Bull. Seismol. Soc. Am. **96,** 879–897 (2006).

2. Abrahamson, N. & Silva, W. Summary of the Abrahamson and Silva NGA Ground-Motion Relations. *Earthq. Spectra* **24,** 67–97 (2008).

3. Jain, S. K., Arlekar, J. N. & Basu, P. C. Empirical attenuation relationships for the Himalayan earthquakes based on Indian strong motion data. in *Proceedings of the sixth international conference on seismic zonation* (2000).

4. Boore, D. M. & Atkinson, G. M. Ground-Motion Prediction Equations for the Average Horizontal Component of PGA, PGV, and 5%-Damped PSA at Spectral Periods between 0.01 s and 10.0 s. *Earthq. Spectra* **24,** 99–138 (2008).

5. Ambraseys, N. N., Douglas, J., Sarma, S. K. & Smit, P. M. Equations for the Estimation of Strong Ground Motions from Shallow Crustal Earthquakes Using Data from Europe and the Middle East: Horizontal Peak Ground Acceleration and Spectral Acceleration. *Bull. Earthq. Eng.* **3,** 1–53 (2005).

6. Atkinson, G. M. & Boore, D. M. Earthquake Ground-Motion Prediction Equations for Eastern North America. *Bull. Seismol. Soc. Am.* **96,** 2181–2205 (2006).

7. Youngs, R. R., Chiou, S.-J., Silva, W. J. & Humphrey, J. R. Strong Ground Motion Attenuation Relationships for Subduction Zone Earthquakes. *Seismol. Res. Lett.* **68,** 58–73 (1997).

8. Lin, T.-L. & Wu, Y.-M. Magnitude estimation using the covered areas of strong ground motion in earthquake early warning. *Geophys. Res. Lett.* **37,** n/a-n/a (2010).

9. Szeliga, W., Hough, S., Martin, S. & Bilham, R. Intensity, Magnitude, Location, and Attenuation in India for Felt Earthquakes since 1762. *Bull. Seismol. Soc. Am.* **100,** 570–584 (2010).

10. Bird, P., Jackson, D. D., Kagan, Y. Y., Kreemer, C. & Stein, R. S. GEAR1: A Global Earthquake Activity Rate Model Constructed from Geodetic Strain Rates and

Smoothed Seismicity. *Bull. Seismol. Soc. Am.* **105,** 2538–2554 (2015).

11. Transparency International. Corruption perception index 2015. (2015). Available at: https://www.transparency.org/cpi2015#downloads. (Accessed: 30th August 2018)

12. United Nations Development Programme. Sub-national Human Development Index (1.0). *Global Data Lab* (2015). Available at: https://hdi.globaldatalab.org/areadata/. (Accessed: 30th August 2018)

13. Wilson, A. M. & Jetz, W. Remotely Sensed High-Resolution Global Cloud Dynamics for Predicting Ecosystem and Biodiversity Distributions. *PLOS Biol.* **14,** e1002415 (2016).
